# Manganese Exposure from Drinking Water and Children’s Classroom Behavior in Bangladesh

**DOI:** 10.1289/ehp.1003397

**Published:** 2011-04-14

**Authors:** Khalid Khan, Pam Factor-Litvak, Gail A. Wasserman, Xinhua Liu, Ershad Ahmed, Faruque Parvez, Vesna Slavkovich, Diane Levy, Jacob Mey, Alexander van Geen, Joseph H. Graziano

**Affiliations:** 1 Department of Environmental Health Sciences, and; 2 Department of Epidemiology, Columbia University Mailman School of Public Health, New York, New York, USA; 3 Department of Psychiatry, College of Physicians and Surgeons, Columbia University, New York, New York, USA; 4 New York State Psychiatric Institute, New York, New York, USA; 5 Department of Biostatistics, Columbia University Mailman School of Public Health, New York, New York; USA; 6 University of Chicago and Columbia University Arsenic Project Office, Mohakhali, Dhaka, Bangladesh; 7 Columbia University Lamont-Doherty Earth Observatory, Palisades, New York, USA

**Keywords:** Bangladesh, children, externalizing behavior, internalizing behavior, manganese, water

## Abstract

Background: Evidence of neurological, cognitive, and neuropsychological effects of manganese (Mn) exposure from drinking water (WMn) in children has generated widespread public health concern. At elevated exposures, Mn has been associated with increased levels of externalizing behaviors, including irritability, aggression, and impulsivity. Little is known about potential effects at lower exposures, especially in children. Moreover, little is known regarding potential interactions between exposure to Mn and other metals, especially arsenic (As).

Objectives: We conducted a cross-sectional study of 201 children to investigate associations of Mn and As in tube well water with classroom behavior among elementary school children, 8–11 years of age, in Araihazar, Bangladesh.

Methods: Data on exposures and behavioral outcomes were collected from the participants at the baseline of an ongoing longitudinal study of child intelligence. Study children were rated by their school teachers on externalizing and internalizing items of classroom behavior using the standardized Child Behavior Checklist-Teacher’s Report Form (CBCL-TRF).

Results: Log-transformed WMn was positively and significantly associated with TRF internalizing [estimated β = 0.82; 95% confidence interval (CI), 0.08–1.56; *p* = 0.03], TRF externalizing (estimated β = 2.59; 95% CI, 0.81–4.37; *p* =0.004), and TRF total scores (estimated β = 3.35; 95% CI, 0.86–5.83; *p* = 0.008) in models that adjusted for log-transformed water arsenic (WAs) and sociodemographic covariates. We also observed a positive monotonic dose–response relationship between WMn and TRF externalizing and TRF total scores among the participants of the study. We did not find any significant associations between WAs and various scales of TRF scores.

Conclusion: These observations reinforce the growing concern regarding the neurotoxicologic effects of WMn in children.

Chronic manganese (Mn) exposure from occupational and environmental sources is known to be associated with an array of neurotoxic health effects. Epidemiologic studies have demonstrated associations between chronic Mn exposure and adverse cognitive and neurologic outcomes in both adults (Bowler et al. 2006, 2007; Ellingsen et al. 2008; Lucchini et al. 1999; Rodriguez-Agudelo et al. 2006) and children (Bouchard et al. 2011; Kim et al. 2009; Menezes-Filho et al. 2009; Takser et al. 2003; Wasserman et al. 2006; Wright et al. 2006). Associations between high levels of Mn exposure and behavioral disturbances have been consistently demonstrated in occupationally exposed adults (Bowler et al. 2007; Laohaudomchok et al. 2010; Lucchini et al. 1999). Findings include increased irritability, anxiety, depression, mood change, somatization, and obsessive and compulsive behavior. Unlike adult reports (Bowler et al. 2007; Laohaudomchok et al. 2010; Lucchini et al. 1999), which have mainly concerned high occupational exposures, the consequences of Mn exposure on children have been investigated primarily in environmental epidemiologic studies at lower exposure levels.

Behavior problems in children are typically grouped into internalizing (anxiety and depression difficulties) and externalizing (behaviors more reflective of insufficient self-control, such as disruptive or conduct problems). An early pilot study (Barlow 1983) reported slightly higher hair manganese (HMn) in 68 hyperactive children compared with the 65 controls, although the study did not take sociodemographic variables into account. In another study of 27 school-age children (Ericson et al. 2007), after adjustment for other potential confounders, prenatal Mn exposure (measured in shed teeth) was associated with reports by parents and teachers of externalizing problems on the Child Behavior Checklist and on teacher-reported disruptive behavior. After adjustment for a limited set of sociodemographic contributors, a study of 47 children reported that externalizing behavioral problems (measured on the Revised Conners’ Teachers and Parents Rating Scales) were significantly elevated in those with higher levels of HMn (Bouchard et al. 2007).

In Bangladesh, both Mn and arsenic (As) in drinking water (WAs) have been recognized as emerging threats to rural public health. To study the health effects of As and Mn, a team of health, earth, and social scientists at Columbia University have been carrying out a number of collaborative projects in Araihazar, Bangladesh, since 2000, including a large cohort study in adults [Health Effects of Arsenic Longitudinal Study (HEALS)] (Ahsan et al. 2006). This large cohort has enabled us to study a population of their children with wide ranges of As and Mn exposures. Through this study we have already documented evidence of adverse impacts of both Mn and As on children’s intelligence (Wasserman et al. 2004, 2006, 2007).

Although prior work suggests that Mn exposure may be more strongly associated with children’s externalizing behavior than with internalizing behavior, the data are inconclusive. To compare associations between both domains of child behavior and exposure, we examined teachers’ reports of behavior problems in a well-characterized group of children, using a sample size sufficiently large to permit these comparisons. We also examined the joint effects of As and Mn on children’s behavior, because synergistic effects of multiple environmental exposures on intelligence have been noted (Kim et al. 2009; Wright et al. 2006).

## Methods

*Overview of the project.* This cross-sectional study is a component of an ongoing, prospective study of child development in Araihazar, Bangladesh. We have previously described the study region and the larger cohort study of adults (Ahsan et al. 2006). The area, with an approximate population of 70,000, is located roughly 25 km northeast of the capital city, Dhaka, within Araihazar Upazilla. Each family in Araihazar typically lives in a house made of tin, mud, hay, or in some cases, concrete. Several houses are clustered together to form a “Bari” representing a small segment of the community (sometimes an extended family).The study region was chosen because it has an extremely wide range of well WAs concentrations (0.1–960 µg/L). In the HEALS study, approximately equal percentages of married men and women, between 18 and 75 years of age, were ultimately recruited, producing a cohort of 11,746 study participants (Ahsan et al. 2006). The children studied here (*n* = 201) are members of the families of cohort participants and are also a subset of those enrolled in an ongoing study of child development. The children in the present report were enrolled in 10 elementary schools located in the HEALS study area.

*Selection of schools and participants.* We recruited 201 children from a larger set of 304 children participating in a separate, clinic-based study of child intelligence. For that study, we generated a list of household wells located within commuting distance of our field clinic using the well WAs and water manganese (WMn) concentrations stored in the HEALS central database. We identified 772 potentially eligible children (8–11 years old) of HEALS participants who were drinking from these household wells. We designated all household wells into one of four groups: *a*) high As, high Mn (As > 10 µg/L and Mn > 400 µg/L); *b*) high As, low Mn; *c*) low As, high Mn; and *d*) low As, low Mn. We continued recruitment until we included approximately 75 in each well category group. The inclusion criteria were the age of the children between 8 and 11 years and who attended schools, did not have chronic illness, and who did not share a home well with other child participants. Of 772 children, 46 could not be located because the family had moved or no one was home at the time of the visit. We also excluded children who were ill, disabled, or had a twin sibling (*n* = 14), who attended schools irregularly (*n* = 40), or whose families had switched wells (*n* = 30) since the initial visit, or who failed to meet the criteria of one eligible child per well (*n* = 51). Parents of 34 children refused to participate in the study, mainly because of the distance and time required to visit the study field clinic. Some children were not eligible because their age could not be determined (*n* = 21) or because they were either younger (*n* = 123) or older (*n* = 102) than the eligible ages. Altogether, 310 children (and their families) agreed to participate in the larger study, 304 of whom (from 37 villages, and relying on 304 unique home wells) were administered the Wechsler Intelligence Scale for Children, 4th edition (WISC-IV) (Wechsler 2004) at our field clinic for the assessment of child intelligence. Urinary and well measures of exposure (both home and school wells) were available on all 304 children, and blood measurements of As and Mn (BAs and BMn, respectively) were available for 300 children. We excluded one child from all analyses because inspection of his hematologic data indicated that he was suffering from a hemoglobinopathy. Enrollment of children in this study, collection of sociodemographic information, and subsequent collection of biological samples from the subjects were conducted during the period of February 2008 and December 2008.

The 304 children attended 30 schools, including 12 schools close to our field clinic. We therefore confined our initial recruitment efforts to the 210 children attending those 12 schools. We approached each principal to invite their participation in the present evaluation of children’s behavior; principals of 10 schools (serving 201 children, taught by 19 teachers) agreed.

*Procedure.* Before conducting this study, we secured approval from the institutional review boards at Columbia University Medical Center and the Bangladesh Medical Research Council and obtained written informed consent from parents as well as child assent. Once parental consent and child assent were obtained, the field team collected sociodemographic information during home visits, at which time well water samples were collected and an appointment was made for the mother and child to visit the field clinic. During their scheduled time at the field clinic, urine and blood samples were collected. We then identified the classroom teachers of the 201 children and obtained their consent for participation in the children’s classroom behavioral assessments. These teachers filled out the Child Behavior Checklist-Teacher’s Report Form (CBCL-TRF) [Achenbach and Rescorla 2001; Achenbach System of Empirically Based Assessment (ASEBA) 2010] during the period of March 2009 and June 2009.

*Translation and training.* All instruments were translated (and back-translated) between Bangla (Bengali) and English. Materials were piloted to ensure teacher and child comprehension. Our study trainer, who had prior experience training professional and community groups in local villages, visited the 10 schools and met with teachers from each school in a group. The trainer and the teachers together read over and discussed the proposed items, and teachers were encouraged to ask questions to clarify any item they found questionable. The trainer provided instructions in how to fill in the TRF. Next, teachers were given a form for each participating child and were asked to complete it within 7 days. Teachers were blind with regard to the household well status of the children.

*Measures.* Teacher characteristics. Characteristics of the teachers were measured by asking them several questions that included their age, number of years in teaching, and their educational qualifications.

Sociodemographic measures. A structured interview was administered during the home visit for each child to collect information on the sociodemographic characteristics of the child. Data on paternal and maternal education and father’s occupation were also collected. Characteristics of the home environment were collected by observation during the interview, including the type of roof, wall, and floor of the house and availability of television and radio. Height, weight, and head and arm circumferences were measured during the child’s visit to our health clinic. During this health clinic visit the mother of the child was also present. At that time, maternal intelligence was measured on the Wechsler Abbreviated Scale of Intelligence (WASI) (Wechsler 1999), a short and reliable measure of intelligence across the age span that comprises two Performance subtests (Block Design and Matrix Reasoning) and two Verbal (Vocabulary and Similarities) subtests.

Well water measurements. Groundwater samples from household wells of the participants were collected in 20-mL polyethylene scintillation vials rinsed several times with the groundwater. Water samples were acidified to 1% with high-purity Optima HCl for at least 48 hr before analysis. This has been shown to ensure re-dissolution of any iron oxides that could have precipitated (van Geen et al. 2007). In the laboratory, all samples were then diluted 1:10 in a solution spiked with ^73^Ge and ^74^Ge for internal drift correction and analyzed by high-resolution inductively coupled plasma mass spectrometry for As and Mn. Further details on field sampling and laboratory analysis procedures are described elsewhere (Cheng et al. 2004; van Geen et al. 2005). For As, the detection limit of the method is typically < 0.2 µg/L, estimated by multiplying the As concentration corresponding to the blank by a factor of 3. The long-term reproducibility determined from consistency standards included with each run averaged 4% (1-sigma) in the 40–500 µg/L range. For Mn, the detection limit of the method is typically < 0.02 mg/L, and the long-term reproducibility averaged 6% in the 0.2–2.0 mg/L range.

Biochemical measurements. As previously described (Wasserman et al. 2006), urinary As (UAs) was measured by graphite furnace atomic absorption spectrophotometry using a PerkinElmer AAnalyst 600 system (PerkinElmer, Shelton, CT, USA) (Nixon et al. 1991). UAs levels were also adjusted for creatinine concentrations, which were analyzed by a colorimetric method based on Jaffe’s reaction (Heinegard and Tiderstrom 1973). Blood hemoglobin levels were determined by standard methods.

Whole venous blood samples were analyzed for BMn, BAs, and blood lead (BPb) concentrations using a Perkin-Elmer Elan DRC II ICP-MS equipped with an AS 93+ auto sampler (Wasserman et al. 2006). A 3-mL EDTA vacutainer of whole blood was thawed, thoroughly mixed, then diluted 50 times with the diluent containing HNO_3_, Triton-X-100, NH_4_OH, and methanol (1%, 0.2%, 0.5%, and 1%, respectively). After centrifugation for 10 min at 3,500 rpm, the supernatant was isolated for analysis. Suitable internal standards were used, which were matched to masses and ionization properties of the analytes. Iridium was used for As, and gallium was used for Mn. After calibrating the instrument, quality control samples were run, that is, blood samples with known analyte concentrations obtained from the Laboratory for ICP-MS Comparison Program (Québec, Canada). Intraprecision coefficients of variation for BMn and BAs were 3.1 and 3.3%, respectively.

*Outcome assessment: child behavior assessment.* The TRF [Achenbach and Rescorla 2001; Achenbach System of Empirically Based Assessment (ASEBA) 2010] is a standardized form by which teachers rate a range of child behaviors, problems, and competencies in youths 4–18 years of age. The TRF contains eight empirically validated scales; of these, we selected two subscales from the internalizing domain (anxious/depressed and withdrawn, e.g., nervous or tense, refuses to talk) and two from the externalizing domain (attention problems and aggressive behavior, e.g., poor school work, screams a lot). The final instrument contained 70 items in four subscales. Teachers rated each child on each item as not true, sometimes true, or often true (scored 0, 1, and 2, respectively). We summed item scores to generate subscale scores and summed subscale scores to generate internalizing, externalizing, and total scores.

*Statistical analyses.* To indicate internal consistency of the TRF items, we calculated Cronbach’s alpha for each TRF subscale, using data from the first child rated by each of the 19 teachers to avoid bias due to within-teacher correlation in the ratings of multiple children from the same teacher. Summary statistics were calculated to describe the sample characteristics. Chi-square test and *t*-test variables were used to detect group difference between children included and excluded from this study in categorical and continuous variables, respectively. Associations between Mn exposure and behavior problems were estimated using repeated measures linear models. This strategy was used to control for within-teacher correlations for the ratings. We first estimated associations between log-transformed exposure variables such as WAs, WMn, urinary creatinine-adjusted (UCr) As, BAs, and BMn, and behavioral outcomes without adjustment for potential confounders. Then we added potential sociodemographic and maternal variables [only body mass index (BMI) was log-transformed] and examined whether they changed the estimated associations between exposure and outcome; variables were retained in the model if there was any substantial change in the association of interest.

We also tested for WAs by WMn interaction by including an interaction term between WAs and WMn and after adjusting for the control variables in the models. Finally, we compared the estimated association between WMn and TRF internalizing behavior with that for the association between WMn and TRF externalizing behavior using the Wald statistic.

## Results

*Sample characteristics.* We compared children who participated in the current study (*n* = 201) with those not included (*n* = 102). No significant differences were found for child’s sex, age, or BMI or for mother’s age, education, or intelligence. Excluded children had slightly and significantly smaller head circumference (49.1 vs. 49.5 cm; *p* = 0.05). Participants had significantly higher WMn (889.2 vs. 397.2 µg/L; *p* < 0.0001) and BMn (15.1 vs. 14.1 µg/L; *p* = 0.03) than did excluded subjects; BPb and BAs concentrations were also higher in participants. On average, teachers of children included in the study were 39 years old and had been teaching for 16.8 years. Sample characteristics including the means, medians, and ranges for the exposure and sociodemographic variables are presented in Table 1.

**Table 1:** Sample characteristics (*n* = 201).

Variable	*n *(%)	Mean ± SD	Median	Range
Male		101 (50.3)						
House construction								
Biomass/hay/mud		4 (2.0)						
Corrugate		171 (85.5)						
Concrete		25 (12.5)						
Child age (years)				9.6 ± 0.8		9.6		8.0–10.9
Months attending school				41.4 ± 16.0		42.0		4.0–78.0
BMI (kg/m^2^)				14.1 ± 1.4		13.9		11.7–25.1
Head circumference (cm)				49.5 ± 1.5		49.5		46.0–53.6
Mother’s education (years)				3.5 ± 3.7		3.0		0.0–16.0
Mother’s age (years)				35.3 ± 6.3		35.0		24.0–52.0
Mother’s WASI raw score				30.9 ± 11.1		30.0		3.0–79.0
Home stimulation				3.6 ± 1.6		4.0		0.0–6.0
Teacher’s years of teaching				16.8 ± 6.8		16.0		6.0–28
Teacher’s age (years)				39.0 ± 6.8		40.0		28.0–55.0
WAs (µg/L)				43.7 ± 67.0		14.0		0.0–371.1
WMn (µg/L)				889.2 ± 783.7		649.5		40.0–3442.5
UAs (µg/L)				81.2 ± 75.2		59.0		6.0–461.0
UCr (mg/dL)				33.9 ± 22.9		28.1		4.9–140.8
UAs (mg/g creatinine)				256.3 ± 181.1		198.3		34.4–1264.6
BAs (µg/L)				5.1 ± 3.3		4.1		0.9–18.0
BMn (µg/L)				15.1 ± 3.9		14.6		6.3–33.9
BPb (µg/L)				120.3 ± 36.4		119.4		36.7–245.7
Hemoglobin (g/dL)				12.5 ± 1.0		12.5		8.4–15.8
Serum ferritin (ng/mL)				34.9 ± 16.4		32.3		2.0–87.5

*Reliability of the outcome measures.* TRF scores are presented in Table 2. The externalizing subscales, that is, aggressive behavior and attention problems, showed higher Cronbach’s alpha values (0.78 and 0.80, respectively) than those for internalizing subscales, although alpha levels for withdrawn/depressed and anxious/depressed were still acceptable (0.65 and 0.51, respectively). TRF internalizing behavior score was also positively and significantly correlated with TRF externalizing behavior score (*r* = 0.58; *p* < 0.0001).

**Table 2:** Classroom behavior characteristics.

Outcome variable	No. of items	Mean scores ± SD	Median	Range
TRF internalizing behavior scores		24		16.7 ± 6.1		16.0		1.0–36.0
Subscale scores								
Anxious/depressed		16		10.3 ± 4.2		10.0		0.0–21.0
Withdrawn/depressed		8		6.4 ± 2.9		6.0		1.0–16.0
TRF externalizing behavior scores		46		31.7 ± 14.0		29.0		4.0–70.0
Subscale scores								
Aggressive behavior		20		9.4 ± 6.5		8.0		1.0–31.0
Attention problems		26		21.6 ± 8.8		21.0		1.0–46.0
TRF total scores		70		47.8 ± 18.3		46.0		11.0–99.0

*Relationships among measures of exposure and outcomes.* Concentrations of WAs, UAs, and BAs were highly correlated (*r* between 0.55 and 0.82) and statistically significant (*p* < 0.0001). WMn was only weakly and nonsignificantly correlated with BMn (*r* = 0.05; *p* = 0.56).

*Associations between sociodemographic and hematologic factors and classroom behavior.* Before the selection of the final models, relationships between the initially selected covariates and the classroom behavior outcome variables were assessed using linear models with repeated measures to adjust for within-teacher correlations. When each of the variables were adjusted for other variables, we observed associations in the expected directions between sociodemographic and maternal factors and classroom behavior scores (Table 3). Boys had significantly higher externalizing behavior scores than girls. Children whose mothers had received more schooling had lower behavior problem scores than those whose mothers had less schooling, although the differences did not achieve statistical significance. Increased BMI was associated with decreased behavioral problem scores.

**Table 3:** Model covariates and TRF scores.

TRF internalizing scores			TRF externalizing scores			TRF total scores		
Outcome variable	Estimated β (95% CI)	*p*-Value	Estimated β (95% CI)	*p*-Value	Estimated β (95% CI)	*p*-Value
Sex (male)		0.43 (–0.94 to 1.80)		0.53		8.68 (4.57 to 12.79)		< 0.0001		9.10 (4.32 to 13.88)		0.0002
Maternal education												
Middle school or higher versus no education		–0.24 (–1.99 to 1.50)		0.79		–2.02 (–6.74 to 2.68)		0.40		–2.16 (–7.61 to 3.29)		0.44
Elementary versus no education		0.92 (–0.95 to 2.80)		0.34		–1.43 (–6.13 to 3.26)		0.55		–0.42 (–6.33 to 5.49)		0.89
BMI*a* (kg/m^2^)		–15.92 (–29.69 to 2.15)		0.02		–27.15 (–64.06 to 9.75)		0.15		–41.64 (–87.92 to 4.63)		0.08
Arm circumference (cm)		0.49 (–0.33 to 1.31)		0.24		2.20 (–0.55 to 4.98)		0.11		2.62 (–0.70 to 5.96)		0.12
**a**BMI was log-transformed.												

*Associations between exposure markers and classroom behavior.* Table 4 presents the relationships between various measures of As and Mn exposure and behavior. After adjustment only for log-transformed WAs, log-transformed WMn was positively and significantly associated with both externalizing and total scores [estimated β = 2.04 and 2.59; 95% confidence interval (CI), 0.26–3.81 and 0.14–5.02; *p* = 0.02 and 0.04, respectively] but nonsignificantly associated with internalizing scores (estimated β = 0.60; 95% CI, –0.11 to 1.32; *p* = 0.10). After adjustment for additional covariates including sex, BMI, maternal education, and arm circumference, the association between WMn and internalizing scores also became significant (estimated β = 0.82; 95% CI, 0.08–1.56; *p* = 0.03). In the finally adjusted models, associations between log-transformed WMn and externalizing and total scores became stronger (estimated β = 2.59 and 3.35; 95% CI, 0.81–4.37 and 0.86–5.83; *p* = 0.004 and 0.008). As the WMn exposure increased, classroom behavior problem scores also increased. In similar models, no significant associations were found between biomarkers of As (BAs, UAs) or Mn (BMn) and any of the classroom behavior outcomes (Table 4). There was a significant difference between the estimated associations between WMn and internalizing and externalizing behaviors (*p* < 0.05), suggesting a stronger association with externalizing behavior.

**Table 4:** Associations between measures of As and Mn exposure and children’s negative classroom behavior scores before and after adjustment.

TRF internalizing scores			TRF externalizing scores			TRF total scores		
Exposure variable (log-transformed)	Estimated β (95% CI)	*p*-Value	Estimated β (95% CI)	*p*-Value	Estimated β (95% CI)	*p*-Value
Water (µg/L) (*n *=**201)												
WAs*a*		–0.31 (–0.68 to 0.06)		0.10		–0.66 (–2.17 to 0.85)		0.39		–1.04 (–2.77 to 0.68)		0.24
WAs*b*		–0.29 (–0.65 to 0.07)		0.12		–0.45 (–1.62 to 0.73)		0.46		–0.78 (–2.18 to 0.62)		0.28
WMn*a*		0.60 (–0.11 to 1.32)		0.10		2.04 (0.26 to 3.81)		0.02		2.59 (0.14 to 5.02)		0.04
WMn*b*		0.82 (0.08 to 1.56)		0.03		2.59 (0.81 to 4.37)		0.004		3.35 (0.86 to 5.83)		0.008
Blood (µg/L) (*n *=**199)												
BAs*a*		–0.92 (–2.44 to 0.60)		0.25		–2.21 (–6.70 to 2.26)		0.33		–3.27 (–9.03 to 2.48)		0.27
BAs*b*		–0.82 (–2.20 to 0.56)		0.24		–2.34 (–6.70 to 2.03)		0.30		–3.25 (–8.81 to 2.31)		0.25
BMn*a*		3.24 (–0.44 to 5.90)		0.08		–4.96 (–11.00 to 1.08)		0.11		–1.76 (–10.39 to 6.86)		0.69
BMn*b*		2.90 (–1.07 to 6.88)		0.15		–2.98 (–9.31 to 3.36)		0.36		–0.03 (–9.36 to 9.29)		0.99
UAs (µg/L) (*n = *199)												
Adjusted for UCr		–0.41 (–1.88 to 1.06)		0.58		–0.85 (–4.86 to 3.15)		0.68		–1.37 (–6.31 to 3.57)		0.59
Adjusted for UCr*c*		–0.37 (–1.68 to 0.95)		0.59		–1.40 (–5.44 to 2.63)		0.50		–1.83 (–6.76 to 3.09)		0.47
Adjusted for UCr + BMn		–0.58 (–1.91 to 0.76)		0.40		–0.66 (–4.66 to 3.35)		0.75		–1.34 (–6.19 to 3.51)		0.59
Adjusted for UCr + BMn		–0.48 (–1.74 to 0.78)		0.45		–1.30 (–5.35 to 2.79)		0.53		–1.86 (–6.74 to 3.01)		0.45
**a**Models adjusted for the other element only. **b**Models adjusted for the other element in addition to sex, maternal education, arm circumference, and log-transformed BMI. **c**Models adjusted for sex, maternal education, arm circumference, and log-transformed BMI. All models were controlled for within-teacher correlations in rating the children.												

We did not observe any statistical interactions between WAs and WMn. The β coefficients for the product term between these two exposure variables were estimated at –0.07 (95% CI, –0.52 to 0.38; *p* = 0.77), –0.71 (95% CI, –1.80 to 0.38; *p* = 0.20), and –0.77 (95% CI, –2.16 to 0.61; *p* = 0.28) for internalizing, externalizing, and total behavior scores, respectively.

**Figure 1:**
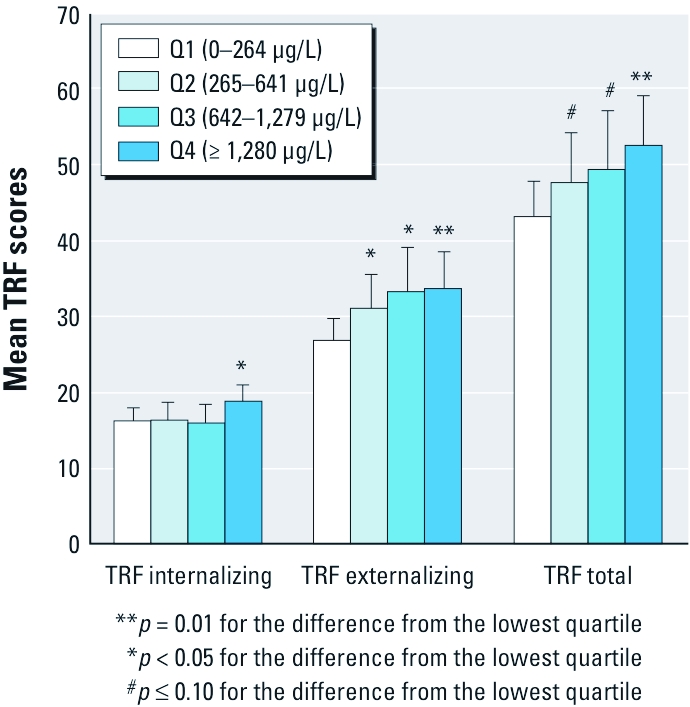
Adjusted mean negative classroom behavior scores by quartiles (Q) of WMn for TRF internalizing, TRF externalizing, and TRF total behaviors. Adjustments were made for sex, maternal education, arm circumference, and BMI and controlling for within-teacher correlations in rating the children. Error bars indicate 95% CI for the quartile-specific estimate.

*Dose–response relationship between well WMn and classroom behavior.* To further describe the dose–response relationships, we categorized WMn into quartiles and examined the adjusted means by quartile (Figure 1). A positive monotonic dose–response relationship was found for externalizing behavior. Compared with the lowest quartile (Q1), estimated βs for Q2, Q3, and Q4 were 4.20 (95% CI, 0.43–7.97); 6.42 (95% CI, 0.80–12.06); and 6.80 (95% CI, 1.42–12.19), respectively. For internalizing behavior, a significant difference was only found between Q1 and Q4, with an estimated β of 2.70 (95% CI, 0.31–5.10). Although the dose–response trend for the total TRF scores was driven largely by the trend for externalizing TRF scores, significant difference between Q1 and Q4 for the internalizing TRF scores also contributed to this trend.

## Discussion

We found positive and significant associations between WMn and both externalizing and internalizing behavior scores, suggesting more problematic behavior as exposure increased. WMn was significantly more strongly related to externalizing compared with internalizing behavior problems. WAs, on the other hand, was not associated with either of the two scales of behavior, and no statistical interaction between WAs and WMn was observed.

*Mn and behavior.* Our findings expand on those of earlier studies of children’s Mn exposure (Bouchard et al. 2007; Ericson et al. 2007), which suggested hyperactive, aggressive, and attention problems (components of the TRF externalizing subscales) but did not directly compare associations for internalizing and externalizing behavior. The average WMn concentration of approximately 900 µg/L in our study (with a median of 650 µg/L and range 40–3443 µg/L) was somewhat higher than a previously conducted WMn study on child intelligence (approximately 800 µg/L) (Wasserman et al. 2006) and substantially higher than another study on children’s cognitive outcomes (approximately 100 µg/L) (Bouchard et al. 2011).

Mn has known impacts on the dopaminergic system (e.g., Tran et al. 2002) as well as on serotonin binding (Velez-Pardo et al. 1995), as demonstrated in animal studies. Compared with controls exposed to low WMn, lower serum levels of serotonin, dopamine, and other neurotransmitters have been found in 92 Chinese children from a region with elevated levels of Mn in drinking water (Zhang et al. 1995). Lower levels of both dopamine and serotonin activity have been associated with a range of child behavior problems including aggressive, violent, and antisocial behaviors (van Goozen et al. 2007; Volavka 1999).

Animal studies of associations between Mn exposure and outcomes such as motor function and cognition have identified brain regions where Mn accumulates. Oral Mn chloride exposure resulted in increased Mn in the striatum, hippocampus, hindbrain, and frontal cortex of rats and nonhuman primates (Dorman et al. 2000; Guilarte et al. 2006; Schneider et al. 2009). The basal ganglia, hippocampus, prefrontal cortex, and amygdala have been implicated in studies of abnormal behavior (Blair 2004; Gorchetchnikov and Grossberg 2007; Saint-Cyr et al. 1995). The amygdala also works as a memory system for more internalizing behavioral events, which may be more emotionally laden, whereas the hippocampus supports identifying the context in which behavioral events take place (McEwen and Gianaros 2010). Abnormalities of the prefrontal cortex and amygdalo–hippocampal complex, as measured by magnetic resonance spectroscopy, have been reported in adult individuals with aggressive behavior and antisocial activities (Critchley et al. 2000). Collectively, this suggests that accumulations of Mn in various areas of the basal ganglia and the frontal cortex could promote externalizing behavior in humans.

We found dose–response associations between WMn and classroom behavior suggesting that those in the first quartile of WMn exposure (< 265 µg/L) differed significantly from those at higher levels. The range of exposure for those within this first quartile falls below the U.S. Environmental Protection Agency (EPA) health advisory level of 300 µg/L for WMn (U.S. EPA 2009). Results suggest, then, that those U.S. children whose drinking water falls outside this range may indeed be at increased risk for behavior problems.

In our earlier work (Wasserman et al. 2006), a study in which levels of WAs were extremely low by design, WMn was associated with child intelligence, whereas BMn was not. Two Canadian studies using HMn reported associations with child IQ (Bouchard et al. 2011) and hyperactive behavior (Bouchard et al. 2007) but did not measure BMn. Another study of Mexican children noted associations with child intelligence for HMn but not BMn (Riojas-Rodriguez et al. 2010). In our current sample, WMn, rather than BMn, was associated with children’s classroom behavior (and WMn and BMn were not significantly correlated). In contrast, studies of adults, even community studies of environmental exposure, have often shown associations between BMn and a range of cognitive functions (Mergler et al. 1999; Santos-Burgoa et al. 2001). BMn may not reliably stand in for the total body burden of Mn (Bouchard et al. 2011), because it seems to reflect recent exposure. Other factors such as duration, frequency, magnitude, and latency of Mn exposure can also potentially impact BMn levels (Smith et al. 2007) Exposure to WMn in our Bangladesh cohort is relatively constant. These inconsistencies suggest that defining the optimal biomarker for Mn exposure remains an open question.

*As and behavior.* We did not observe any significant association between any measure of As exposure and classroom behavior. Adding WAs to models did not change the pattern of associations between classroom behavior and either WMn or sociodemographic variables; we observed no interaction between WMn and WAs. Although our research group has reported decrements in intelligence associated with As exposure in children (Wasserman et al. 2004, 2007), we have not been able to identify reports of associations between As exposure and behavioral problems.

*Limitations.* The cross-sectional nature of this study hinders cause–effect inferences. However, we argue that the direction of the relation is exposure to outcome, because it is unlikely that behavior will influence well usage. Additionally, we examined a narrow age range; we do not know whether the behavior-related problems could be detected at younger ages or whether the problems intensify at older ages. All participating children (and schools) have now been provided with access to deep wells that are much lower in As and Mn. We continue to follow the cohort and will reassess behavior, intelligence, and motor function longitudinally.

Our study was conducted in a rural area of Bangladesh, which is relatively well developed and where families are primarily middle class to lower middle class. Our sample, then, may not represent all of Bangladesh, and results may be generalizable only to communities with similar rural sociodemographic characteristics.

Unmeasured teachers’ biases might have evolved from teacher–child interactions at the family level in rural Bangladesh sociocultural context. In most cases, teachers in village schools know the family background of the children very well and may occasionally have friendly or strained relationships with the child’s family.

Finally, the large difference in WMn concentrations between children included and children excluded from this study deserves comment (889.2 vs. 397.2 µg/L, respectively). Our HEALS study region is roughly 25 km^2^ in size. By chance, families with relatively lower WMn more commonly reside in the more rural NW corner of this region, farthest from our field clinic/headquarters, and schools in that part of our study area are smaller and more scattered; these schools were therefore excluded. This resulted in a higher overall level of WMn exposure among those studied here, as children attending the most distant schools were excluded.

## Conclusion

Our study is unique in several ways. First, by using a well-standardized measure of child behavior problems that assesses both externalizing and internalizing behavior, we had the opportunity to examine specificity in the exposure/behavior problems association. These scales demonstrated good to excellent reliability and validity in the current population. Second, we took a diverse set of sociodemographic variables into account while examining the relationship between exposure and childhood behavioral problems.

From a global public health point of view, our findings serve as an indication of concern for countries where drinking water may be contaminated with high Mn levels. In the United States, where 5.2% of household wells contain > 300 µg/L Mn (DeSimone et al. 2009), large numbers of children may be at a risk of developing behavioral problems.
